# On the role of *AtDMC1*, *AtRAD51* and its paralogs during *Arabidopsis* meiosis

**DOI:** 10.3389/fpls.2014.00023

**Published:** 2014-02-17

**Authors:** Mónica Pradillo, Javier Varas, Cecilia Oliver, Juan L. Santos

**Affiliations:** Departamento de Genética, Facultad de Biología, Universidad Complutense de MadridMadrid, Spain

**Keywords:** *AtRAD51*, *AtDMC1*, *AtRAD51C*, *AtXRCC3*, meiosis, homologous recombination, *Arabidopsis*

## Abstract

Meiotic recombination plays a critical role in achieving accurate chromosome segregation and increasing genetic diversity. Many studies, mostly in yeast, have provided important insights into the coordination and interplay between the proteins involved in the homologous recombination pathway, especially the recombinase RAD51 and the meiosis-specific DMC1. Here we summarize the current progresses on the function of both recombinases and the CX3 complex encoded by *AtRAD51 *paralogs, in the plant model species *Arabidopsis thaliana*. Similarities and differences respect to the function of these proteins in other organisms are also indicated.

## INTRODUCTION

More than 10 years ago the *Arabidopsis* nuclear genome DNA sequence was published (*Arabidopsis* Genome Initiative, 2000). Afterward, this species has emerged as the most important experimental system in plant science. Discoveries made in *Arabidopsis* during the past decade have even led to the understanding or recognition of molecular processes in other organisms, including humans (Jones et al., 2008). Thus, *Arabidopsis* has now become an indispensable tool to understand basic cellular processes such as meiosis, a specialized type of cell division by which sexually reproducing eukaryotes maintain their chromosome number across generations.

During meiosis two rounds of cell division follow a single round of DNA replication to produce haploid gametes. The physical connections between homologous chromosomes (chiasmata) produced as consequence of reciprocal recombination events [crossovers (COs)], in combination with sister chromatid arm cohesion, are responsible for the correct bi-orientation of bivalents at metaphase I and the subsequent segregation of a complete set of chromosomes at anaphase I. Sister chromatids separate at the second division generating haploid gametes. Fusion of gametes at fertilization restores the diploid chromosome number of the species and initiates zygote development. Most knowledge of this process, specifically on recombination mechanisms, derives from studies in *Saccharomyces cerevisiae *and *Schizosaccharomyces pombe*. However, during the past decade, analysis of meiotic homologous recombination (HR) has been boosted by the combination of forward (from phenotype to genotype) and reverse (from genotype to phenotype) genetics in *Arabidopsis* (Mercier and Grelon, 2008; Osman et al., 2011). Another approaches to identify meiotic genes in this species have consisted on evaluation of transcriptome profiling from flower buds, anthers and meiocytes (Schmid et al., 2005; Zhang et al., 2005; Wijeratne et al., 2007; Chen et al., 2010; Libeau et al., 2011; Yang et al., 2011), and the proteome profiling from anthers of the close relative *Brassica oleracea* (Sánchez-Morán et al., 2005). On these grounds, numerous data on the mechanism, specificity and regulation of meiosis have been accumulated. Moreover, plants have an advantage over other eukaryotes because their meiosis is completed (not arrested) in mutants defective in recombination, synapsis or segregation (Jones and Franklin, 2008; Wijnker and Schnittger, 2013). The present review analyzes the current knowledge about the process of meiotic HR in *Arabidopsis*, and is mainly focused on the role of AtRAD51 and AtDMC1 and the CX3 complex, which is encoded by *AtRAD51* paralogs.

## INITIATION OF MEIOTIC RECOMBINATION IN *Arabidopsis*

One feature that distinguishes model organisms in relation to HR is the link between recombination and pairing. In plants, as well as in yeasts, mice and humans, and unlike fruit flies and nematodes, initiation of the recombination process is essential for accurate homologous interactions that match homologous together (Dernburg et al., 1998; McKim et al., 1998; Gerton and Hawley, 2005). HR is a process by which DNA sequences are exchanged between homologous sequences (homologous chromosomes or sister chromatids). In meiosis HR is a critical process required to repair the DNA double-strand breaks (DSBs) produced at prophase I. DSB formation is catalyzed by Spo11, which is related to the Top6A subunit of archaeal type IIB topoisomerases. It is a meiosis specific protein conserved in almost all eukaryotes (Bergerat et al., 1997; Keeney, 2001). Although it seems to be lost in *Dictyostelium discoideum* (Malik et al., 2007), the function of Spo11 initiating meiotic recombination seems to be universal (Dernburg et al., 1998; McKim and Hayashi-Hagihara, 1998; Romanienko and Camerini-Otero, 1999; Celerin et al., 2000; Grelon et al., 2001; Lichten, 2001; see de Massy, 2013 for a more recent review). In mice and humans there are two isoforms of SPO11, SPO11α and SPO11β. The latter one is responsible for producing most DSBs (Romanienko and Camerini-Otero, 1999; Bellani et al., 2010). In plants several *SPO11* paralogs have been identified: there are five genes in *Oryza sativa* and three genes in *Arabidopsis*. In rice, only *OsSPO11-1* and *OsSPO11-4* are needed for meiosis (Yu et al., 2010; An et al., 2011). In *Arabidopsis*, *AtSPO11-1 *and *AtSPO11-2 *play a role in meiotic HR whereas *AtSPO11-3 *is involved in DNA endoreduplication (Hartung and Puchta, 2000; Grelon et al., 2001; Hartung et al., 2002; Sugimoto-Shirasu et al., 2002; Stacey et al., 2006). There is a relatively low sequence similarity between the genes (20–30%), suggesting they are products of an ancient duplication. AtSPO11-1 and AtSPO11-2 display a non-redundant function in DSB formation and it has been suggested that both proteins could work coordinately, forming a heterodimeric complex or interacting reciprocally to get wild-type levels of DSBs (Stacey et al., 2006). The double mutant *Atspo11-1 Atspo11-2* does not differ phenotypically from the respective single ones, hence both proteins would be required for the same step in the pathway. Hartung et al. (2007a) even proposed that each DNA strand could be broken by different AtSPO11 proteins. However, Shingu et al. (2010) have described a multimeric active form of AtSPO11-1.

In *Saccharomyces cerevisiae* the meiotic DSB formation depends on Spo11 and at least nine more proteins (Keeney, 2001, 2008) and the same occurs in *Schizosaccharomyces pombe* for Rec12 (*Rec12* is the *Spo11* ortholog in this species; Davis and Smith, 2001; Young et al., 2004). Only some of these proteins are conserved in *Arabidopsis*, but they do not participate in DSB formation (Edlinger and Schlögelhofer, 2011). For example, the tight dependence of Spo11 cleave on the MRX complex is not conserved in *Arabidopsis *(Gallego et al., 2001; Bleuyard et al., 2004; Puizina et al., 2004; Akutsu et al., 2007). Likewise, *Ski8* orthologs are required for DSB formation in *S. cerevisiae*, *S. pombe* and *Sordaria*, but in *Arabidopsis* *Ski8* is dispensable for meiosis (Jolivet et al., 2006). On the other hand, forward genetic approaches have led to identification of other genes necessary for DSB formation in *Arabidopsis*: *AtPRD1*, *AtPRD2*, and *AtPRD3* (De Muyt et al., 2007, 2009). These authors suggested that AtPRD1 could contribute to generate an asymmetry in DSB processing. Although an *AtPRD1* ortolog has not been found in fungi, there is a similarity between *AtPRD1* and *MEI1* and between *AtPRD2* and *MEI4*. Both, MEI1 and MEI4, are involved in the first steps of mammalian meiosis (Libby et al., 2003; Kumar et al., 2010). Also, *PAIR1*, the rice ortholog or *AtPRD3*, is required for DSB formation (Nonomura et al., 2004). Another *Arabidopsis* DSB forming protein recently discovered is *Arabidopsis thaliana* DSB formation (AtDFO), a plant-specific protein without any known conserved domain (Zhang et al., 2012). These findings suggest that, although the DSB formation by Spo11 is well conserved, there are significant mechanistic differences in the regulation of this process in different organisms.

## REPAIR OF DOUBLE-STRAND BREAKS BY HOMOLOGOUS RECOMBINATION: CROSSOVERS AND NON-CROSSOVER

**FIGURE 1:**
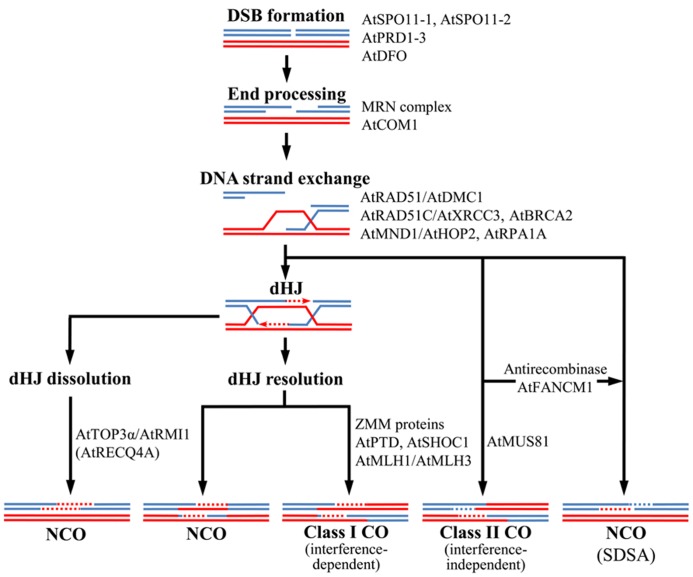
** Model for crossover (CO) and non-crossover (NCO) formation in *Arabidopsis *meiosis.** Double-strand breaks (DSBs) are formed and their 5′ ends processed to generate 3′-ended single-stranded DNA (ssDNA). One of these ends invades the homologous duplex DNA, giving raise a D loop intermediate. If the second end is captured and the broken DNA strands are ligated, a double Holliday junction (dHJ) is formed. This dHJ can be dissolved as a NCO or resolved to form either a NCO or a class I CO, sensitive to interference. Alternatively, the D loop can be processed to generate a class II CO (insensitive to interference, this class includes nearly 15% of total COs). Presumably most NCOs arise via synthesis-dependent strand annealing (SDSA) and they are produced when the D loop is dissociated before the second end capture. Main proteins involved in *Arabidopsis *meiotic recombination are shown (see text for a more detailed explanation).

A HR event between two homologous sequences can result in either CO or non-CO (NCO). In COs there is a reciprocal exchange of alleles flanking the DSB position, the formation of at least one CO (the obligatory chiasma) is necessary to hold homologous chromosomes together until their segregation to opposite poles at anaphase I. In NCOs, which are also formed associated to CO events, flanking alleles maintain their original linkage. The current model to explain meiotic HR was proposed in yeast by Bishop and Zickler (2004). It broadly concurs with a previous model (Szostak et al., 1983; Sun et al., 1989) modified by Stahl (1994), with the exception that the CO/NCO decision is made earlier, before double Holliday junction (dHJ) resolution and synaptonemal complex (SC) formation (Allers and Lichten, 2001). This model is apparently suitable for *Arabidopsis* (Ma, 2006; Sanchez-Moran et al., 2008). **Figure 1** illustrates the main steps of the *Arabidopsis* meiotic recombination pathway and the proteins involved. In this model, NCOs may be produced by synthesis-dependent strand-annealing (SDSA), which is a mechanism involving strand invasion, DNA synthesis and strand ejection that does not involve HJs. However, SDSA is not the only way to produce NCO during meiosis in this species. Thus, AtTOP3α and AtRMI1, which constitute a complex with a RecQ helicase, contribute to dissolve dHJs and generate some NCO products (Chelysheva et al., 2008; Hartung et al., 2008). In yeast, the helicase Sgs1 is the master controller of meiotic recombination and determines whether a recombination intermediate becomes either a NCO product or is directed toward a pathway that ultimately ends up as a CO (De Muyt et al., 2012; Zakharyevich et al., 2012). Expression of *AtRECQ4A* in yeast, the *Arabidopsis Sgs1* ortholog, resulted in full suppression of the *sgs1* mutant phenotype, indicating that both proteins apparently play the same function (Bagherieh-Najjar et al., 2005; Hartung et al., 2007b). However, AtRECQ4A does not seem to play a prevalent role on CO formation (Higgins et al., 2011). In addition, DNA translocase Fanconi anemia complementation group M (FANCM) acts as an antirecombinase, processing meiotic DSB repair intermediates and driving them toward NCO, constraining CO formation (Crismani et al., 2012; Knoll et al., 2012).

Crossovers are originated by two different ways: class I COs are interference sensitive (subject to nearby COs) and dependent on the ZMM proteins (Börner et al., 2004,), whereas class II COs are randomly distributed and dependent on Mus81 and Mms4 proteins for their formation (de los Santos et al., 2003). Here the word “interference” refers to positive interference, which is the spacing of events that departs from a random distribution. Although both CO classes are initiated by DSBs, they are generated through different intermediates: single Holliday Junctions (sHJs) for class II COs and dHJs for class I COs. Different model organisms are dependent on both CO classes in a different way: in fission yeast there are not interference sensitive COs (Osman et al., 2003; Smith et al., 2003; Cromie et al., 2006), whereas in *Caenorhabditis elegans *all COs show interference (Zalevsky et al., 1999; Hillers and Villeneuve, 2003). An intermediate situation is found in budding yeast, mammals and *Arabidopsis*, whose proteins required for both CO classes are present (Copenhaver et al., 2002; de los Santos et al., 2003; Housworth and Stahl, 2003; Berchowitz et al., 2007; Higgins et al., 2008a, b; Holloway et al., 2008). In *Drosophila melanogaster* all COs are produced by a third CO pathway (Yildiz et al., 2002) and associated with interference (McPeek and Speed, 1995). Although it is unclear why some species have conserved two CO pathways, this is a general feature in plant kingdom (Tuskan et al., 2006; Mimida et al., 2007; Paterson et al., 2009). Furthermore, it is possible that a third CO pathway coexists with those previously mentioned, since some COs still occurs when the main proteins involved in class I and II COs are eliminated in yeast (de los Santos et al., 2003; Argueso et al., 2004) and *Arabidopsis* (Berchowitz et al., 2007; Higgins et al., 2008a).

It is noteworthy that there are differences in the DSBs/COs ratio among species (Serrentino and Borde, 2012). Neither physical genome size nor chromosome number can explain these differences. Budding yeast shows only about two times more DSBs than COs (Mancera et al., 2008). However, in *Arabidopsis* and mammals, precursor sites of recombination events are much more numerous than COs, suggesting that DSBs are mostly repair to give NCOs (Baudat and de Massy, 2007; Sanchez-Moran et al., 2007). In *Arabidopsis*, the number of NCOs per meiosis detected by different approaches [next generation sequencing (NGS); tetrad analysis; pollen typing] is, in general, very low (ranging from one to six; Lu et al., 2012; Sun et al., 2012; Drouaud et al., 2013; Wijnker et al., 2013). Since there are 120–140 DSBs in a single meiosis, as indicated by the number of AtDMC1 foci (Sanchez-Moran et al., 2007), and COs vary from 7 to 11 depending on the accession studied (Sanchez-Moran et al., 2002; López et al., 2012), one possible explanation for this discrepancy is inter-sister repair of DSBs. However, the relative importance of the sister as template during meiosis is totally unknown in *Arabidopsis*, and it seems to occur at a low frequency in other organisms (Pradillo and Santos, 2011). Other reasons for the low gene conversion rates detected could be either short NCO tracts (<100 bp), that would be undetectable because they do not convert a single nucleotide polymorphism (SNP), or the preferential repair of heteroduplexes to parental genotypes (Lu et al., 2012; Wijnker et al., 2013). On the contrary, another study using NGS approaches has concluded that between 90 and 99% of recombination events are due to gene conversion (Yang et al., 2012). Additional research should be addressed to confirm the discrepancy between these data.

## RAD51, DMC1, AND DNA STRAND EXCHANGE

Once generated by Spo11, resection of the DSB proceeds in a 5′ -3′ direction, resulting in 3′ single-stranded tails that are required for the following step in the meiotic HR process. Following this resection, 3′ single-stranded DNA (ssDNA) tails are generated around the DSB site. In this context, HR occurs with a partner DNA intact duplex in a strand exchange reaction catalyzed by Rad51 (which functions during HR in all cell types) and Dmc1 (the meiosis-specific recombinase) to get joint molecule intermediates (Neale and Keeney, 2006). These DNA recombinases bind to the extruded ssDNA tails, to form presynaptic filaments. Since meiotic HR possesses particular features different to mitotic HR, the preference for interactions between homologs instead of sisters among others, it has been proposed that Dmc1 is essential to carry out these unique peculiarities. Even, based on the asymmetric DSB processing, some authors have hypothesized that Rad51 and Dmc1 could be loaded differentially on the distinct DNA ends (Shinohara et al., 2000; Hunter and Kleckner, 2001; Neale et al., 2005). However, despite the numerous investigations, the exactly way both recombinases work is unknown. Furthermore, there are organisms that lack a Dmc1 ortholog, like *Drosophila melanogaster *and *C. elegans*. Curiously, these species achieve synapsis independently of DSBs and display COs subject to interference. This finding led Copenhaver et al. (2002) to propose that organisms possessing Dmc1 use Dmc1-mediated non-interfering COs to achieve synapsis, whereas synapsis occurs by a recombination-independent route in those lacking this protein. However, *Neurospora* lacks both non-interfering COs and Dmc1, but requires DSBs for synapsis (Bowring et al., 2006). On the other hand, *Arabidopsis* requires the early recombination function of SPO11 proteins to achieve synapsis (Grelon et al., 2001; Stacey et al., 2006), grouping with yeast, mouse, grasshopper and *Coprinus*, and possesses a *DMC1 *homolog (Kleckner, 1996; Celerin et al., 2000; Mahadevaiah et al., 2001; Viera et al., 2004).

In budding yeast, the relative importance of Rad51 and Dmc1 in promoting meiotic interhomolog instead of intersister recombination has been matter of debate in the last years. Mutants defective in Rad51 present decreased recombination in meiosis, with reduced spore viability (Petes et al., 1991), and fail to form Dmc1 foci (Bishop, 1994). Mutants defective in Dmc1 show defects in meiotic recombination and accumulate recombination intermediates, showing problems to form normal SCs and arresting at late prophase I (Bishop, 1994). However, Bishop et al. (1999) and Tsubouchi and Roeder (2003) demonstrated that high levels of interhomolog recombination could be achieved in the absence of Dmc1 upon either overexpression of *Rad51* or its stimulating partner *Rad54*. These results revealed that both recombinases are able to catalyze interhomolog recombination during meiosis. Nevertheless, Rad51 strand exchange capacity during meiosis is specifically shut down by meiosis-specific factor Hed1 and Rad54 phosphorylation in such a way that interhomolog recombination is then mediated exclusively by Dmc1 (Tsubouchi and Roeder, 2006; Niu et al., 2009; Busygina et al., 2012). Indeed, Cloud et al. (2012) have recently reported that only the strand exchange activity of Dmc1, and not that of Rad51, is necessary for meiotic recombination. Thus, Rad51 would be a Dmc1 accessory factor which contributes to homolog bias independently of its strand exchange activity. However, exchange activity of Rad51 may also be relevant as a fail-safe when Dmc1 fails. To add more complexity to this landscape Hong et al. (2013) have reported that, contrarily it was though, the default option for recombination is homolog bias, independently whether strand exchange is promoted by either Dmc1 or Rad51. These authors proposed a model in which the role of Rad51/Dmc1 interplay for the establishment of homolog bias is to counteract a role of Rec8 that promotes sister bias. Both recombinases would contribute to homolog bias: *rad51Δ* exhibits sister bias and the same occur in the double mutant *hed1Δ dmc1Δ* (*hed1Δ* mutation permits Rad51 to carry out strand exchange when Dmc1 is absent). These authors also provide evidences for Dmc1 being an inhibitor of Rad51 activity. Recently, Lao et al. (2013) have reported a similar inhibitory function of Dmc1.

Curiously, in fission yeast the preferential polarities of Holliday junction branch migration driven by Rad51 and Dmc1 are different (Murayama et al., 2008, 2011). Despite similarities in protein structure and reaction features, Dmc1 promotes exchange in the 5′-to-3′ direction relative to the ssDNA region of the DNA substrate, while Rad51 does it in the 3′-to-5′ direction. These differences may be important in the pathway from HR intermediate formation to CO production. Murayama et al. (2011) also proposed a role for Dmc1 in the second end capture of the DSB site.

What do we know about the function of RAD51 and DMC1 in plants? The *RAD51 *gene exists as a single copy in tomato and *Arabidopsis*, while maize has two copies (Stassen et al., 1997; Doutriaux et al., 1998; Franklin et al., 1999; Li et al., 2004). *DMC1 *gene sequences have been reported in a few plant species. While *Arabidopsis* genomes contain one copy of this gene, rice has two copies, *OsDMC1A *and *OsDMC1B* (Klimyuk and Jones, 1997; Kathiresan et al., 2002). On the contrary, there are three expressed copies of each of the *TaRAD51 *and *TaDMC1 *homoeologues in bread wheat (Devisetty et al., 2010). However, most knowledge about the function of these genes in plants has been deduced from the study of the corresponding *Arabidopsis* mutants. Whereas *dmc1* budding yeast mutants show an accumulation of unprocessed DSBs and form abnormal SCs (Bishop et al., 1992), the *dmc1* mutant phenotype is distinct in *Arabidopsis*.* Atdmc1* fails to undergo synapsis and displays ten unfragmented univalent chromosomes at metaphase I (Couteau et al., 1999). Thus, in this mutant the DSBs are repaired efficiently from the intact duplexes of sister chromatids, probably by AtRAD51. According with this, *AtRAD51 *expression, induced in young flower buds, is increased in both homozygous and heterozygous *Atdmc1 *plants when compared with the wild-type (Couteau et al., 1999). In contrast to *Atdmc1*, the *Atrad51* mutant exhibits meiotic defects in pairing and synapsis, as well as a severe AtSPO11-dependent chromosome fragmentation (Li et al., 2004). However, in both mutants the vegetative development is not affected, indicating that the function of both recombinases is dispensable for processing mitotic DSBs under normal growth conditions. The different phenotype of *Atdmc1* and *Atrad51* indicates that AtDMC1 could be primarily responsible for DSB repair using the homologous chromosome as a template. In contrast, AtRAD51 would repair DSBs using a sister chromatid as a template. This model has also been proposed in the protist *Tetrahymena*, in which in the absence of DMC1, efficient RAD51-dependent repair take place, but COs are suppressed (Howard-Till et al., 2011).

**FIGURE 2:**
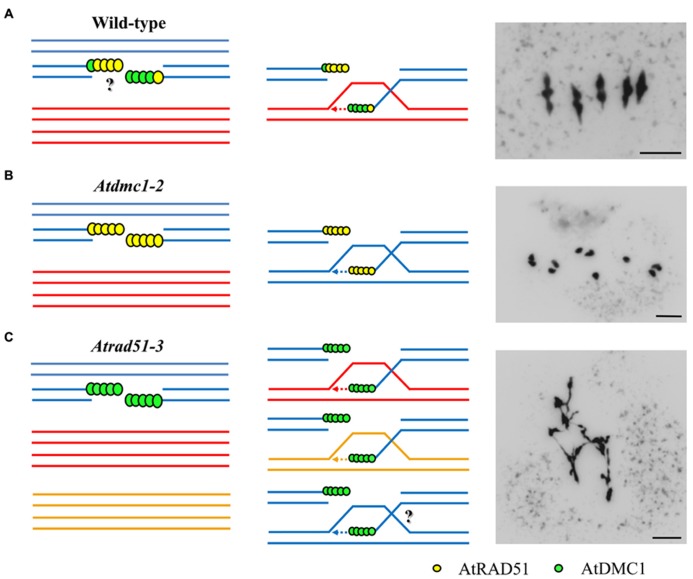
** Model for the interplay between AtRAD51 and AtDMC1 during homologous meiotic recombination in *Arabidopsis*. (A)** During WT meiosis, it is not known if AtRAD51 (yellow) and AtDMC1 (green) are loaded asymmetrically at both DNA ends. According to several studies (see text for details) the AtDMC1 nucleofilament would be involved in the first strand invasion, whereas AtRAD51 nucleofilament would be responsible in checking homology during the second end invasion. However, the existence of mixed nucleofilaments could not be ruled out. Five bivalents are observed at metaphase I. **(B)** In the *Atdmc1-2* mutant (Pradillo et al., 2012), all the nucleofilaments are constituted by AtRAD51. DSBs are repaired using the sister chromatid as template. The result is the formation of ten univalents at metaphase I, and complete absence of chromosome fragmentation. **(C)** In the *Atrad51-3* mutant (knockout mutant; Pradillo et al., 2012), all the nucleofilaments are constituted by AtDMC1. The entangled mass of chromosomes observed at metaphase I could indicate recombination between homologous and non-homologous chromosomes. Sister chromatid exchanges could happen. Red and blue lines represent homologous chromatids. Orange lines represent chromatids from a non-homologous chromosome. Bars represent 5 μm.

In this context, AtBRCA2 could be involved in the interplay between both recombinases, since it is required for the proper recruitment of both AtRAD51 and AtDMC1 (Seeliger et al., 2012). Likewise, an axis-associated protein related to the yeast Hop1 (ASY1), could play a role in coordinating the activity of the recombinases to favor interhomolog recombination in *Arabidopsis *(Sanchez-Moran et al., 2007). Thus, ASY1 has a differential effect on the localization of AtRAD51 and AtDMC1. AtRAD51 localization is independent on ASY1, but AtDMC1 fails to form a stable association with chromatin when ASY1 is absent. The disruption of AtDMC1 localization in *asy1 *mutant produces severe defects on chromosome alignment, synapsis and recombination, although all DSBs are repaired and chromosomes do not show fragmentation. The same occurs in the mutant *sds*, defective for a cyclin-like protein, which also shows defects in AtDMC1 localization (De Muyt et al., 2009). On the other hand, there is a slightly asynchrony in the chromatin loading of AtRAD51 and AtDMC1. The accumulation of AtDMC1 precedes that of AtRAD51 (Sanchez-Moran et al., 2007). This fact could reflect the asymmetry in the loading of both proteins to the resected DSBs, like has also been proposed in yeast (Hunter and Kleckner, 2001). Indeed, Kurzbauer et al. (2012) have reported that AtRAD51 and AtDMC1 do not colocalize during any meiotic stage, in concordance with the hypothesis that the recombinases occupy opposite DNA ends at a DSB. These results, together with the meiotic phenotype of these mutants, lead to suggest that the AtDMC1-end would be chiefly responsible for the initial strand invasion of the homolog, whereas the AtRAD51-end would be captured at a later stage (**Figure 2**). However, Kurzbauer et al. (2012) have proposed that the AtDMC1-end could be able to repair from both sister chromatids and homologous chromosomes, the strand exchange activity of RAD51 being dispensable for meiosis. They inferred this conclusion from the meiotic phenotype of the double mutant *atr rad51*, in which meiotic DSBs are repaired to a certain extent, even resulting in bivalent formation in some meiocytes. They also confirmed that *Atrad51* is defective in AtDMC1 loading, whereas AtRAD51 foci numbers are not altered in *Atdmc1*. It would explain the meiotic phenotype of the corresponding mutants. This fact does not occur in other species such as *Tetrahymena*, in which DMC1 focus formation is independent of the presence of RAD51 (Howard-Till et al., 2011). Kurzbauer et al. (2012) also argue that ATR has a pivotal role in the meiotic program and propose a model in which AtRAD51 loading attenuates ATR signaling, allowing AtDMC1 loading (see Figure 5 in Kurzbauer et al., 2012). In this model ASY1 would inhibit AtDMC1-end for DSB repair using sister chromatids as template. This model would explain why the DNA repair of meiotic DSBs is even more efficient in the triple mutant *atr rad51 asy1* than in the double mutant *atr rad51*. The ability to AtDMC1 to repair using the sister has also been proved in a haploid context (Crismani et al., 2013).

On the other hand, Da Ines et al. (2013a) have reported that AtRAD51 would just play a supporting role in meiotic recombination, as well as in yeast. This assertion is based on the effect of a RAD51-GFP fusion protein that retains ability to assemble at DSBs but lacks strand exchange activity. This protein is capable to repair the meiotic chromosomal fragmentation and sterility showed by *Atrad51*, without an increase of COs. However, this complementation is not possible in the double mutant *Atrad51 Atdmc1*, being fully dependent on the presence of AtDMC1. Also, another similarity between plant and yeast has been found by Uanschou et al. (2013). These authors have demonstrated that in the absence of AtDMC1, AtMND1/AtHOP2, an essential complex during HR, is dispensable for AtRAD51-mediated intersister DNA repair. However, in the presence of AtDMC1, a minimal amount of functional AtMND1/AtHOP2 is indispensable to drive intersister DNA repair, suggesting that AtDMC1 is a negative regulator of AtRAD51 during meiosis. This negative regulation would be critical for the establishment of AtDMC1-dependent interhomolog connections.

Taking into account all the observations mentioned above, it is time to ask for the role of RAD51 during plant meiotic recombination. RAD51 is a component of the early recombination nodules, required for homology searching and synapsis in lily (Anderson et al., 1997, 2001). In maize, Franklin et al. (1999) observed that the number of RAD51 foci exceeds the number of COs. This finding led to the suggestion that these extra foci may be involved in homology search. Furthermore, Pawlowski et al. (2003) pointed out that completion of homologous pairing is necessary for the removal of RAD51 from chromosomes. In addition, maize plants deficient in RAD51 function exhibit unprocessed DSBs, as well as non-homologous synapsis and chiasmata between non-homologous chromosomes (Li et al., 2007). A knock-down *Atrad51* mutant displays a similar phenotype: multivalents and well-defined homologous and non-homologous bivalents (Pradillo et al., 2012). This mutant also presents less fragmentation than the knock-out, suggesting that somehow DSBs are being processed by HR, although a critical level of AtRAD51 is required to ensure the fidelity of HR during interchromosomal exchanges. This work highlighted, for first time in *Arabidopsis*, that in addition to its strand exchange activity, AtRAD51 could also be required to ensure the fidelity of HR in the interchromosomal exchanges initiated by AtDMC1. Still further, Shinohara and Shinohara (2013) have recently described a role for Rad51 in the suppression of meiotic ectopic recombination in *Saccharomyces cerevisiae*. In summary, AtRAD51 seems to play two different, although interrelated, meiotic functions: one lying in its strand exchange activity and other lying in helping AtDMC1 to promote CO and to guarantee a faithful recombination. The former appears to be dispensable during the meiotic process as it has been revealed by recent studies (Kurzbauer et al., 2012; Da Ines et al., 2013a). However, in this landscape many questions remain unanswered, including the role of *AtRAD51 *paralogs.

## RAD51 PARALOGS: THE CX3 COMPLEX

In addition to *RAD51 *and *DMC1*, *Arabidopsis*, in common with vertebrates, possesses five *RAD51* paralogs: *RAD51B*, *RAD51C*, *RAD51D*, *XRCC2*, and *XRCC3 *(Osakabe et al., 2002; Bleuyard et al., 2005). Yeast two-hybrid and co-immunoprecipitation studies have shown that proteins coded by these *RAD51* paralogs form two complexes in vertebrates: CX3 (RAD51C/XRCC3) and BCDX2 (RAD51B/C/D/XRCC2; Schild et al., 2000; Masson et al., 2001; Liu et al., 2002). Presumably these genes arose by gene duplication and acquired new functions during evolution, their exact role being not fully understood to date. Both complexes are involved in DNA repair, but only CX3 plays essential roles in meiotic HR (Bleuyard and White, 2004; Li et al., 2005; Osakabe et al., 2005). In mammals, biochemical studies have detected that in the CX3 complex, RAD51C facilitates homolog pairing, whereas XRCC3 contributes to the preferential binding of the complex to ssDNAs (Kurumizaka et al., 2001), and both proteins promote the loading of RAD51 onto DNA (Bishop et al., 1998; Masson et al., 2001; Wiese et al., 2002). It has also been proposed that CX3 functions in late stages of HR pathway, resolving Holidays junctions (Liu et al., 2004, 2007; Sharan and Kuznetsov, 2007). Recently, Chun et al. (2013) have demonstrated in human cells that in response to DNA damage, BCDX2 and CX3 complexes act upstream and downstream of RAD51 recruitment, respectively, and both are epistatic with BRCA2.

In yeast, *Rad51* paralogs are named *Rad55* and *Rad57* and the corresponding proteins form a heterodimeric complex which associates with Rad51 nucleofilament. DSB repair defects and DNA damage sensitivity generated by mutations in these genes can be rescued by *rad51* gain-of-function alleles (Fortin and Symington, 2002). Both proteins, Rad55 and Rad57, are not functionally equivalent since a mutation in the gene *Rad55* has a much stronger effect respect to sensitivity to irradiation (Johnson and Symington, 1995). It is noteworthy that either *rad55Δ* or *rad57Δ* reduce interhomolog bias during meiotic recombination (Schwacha and Kleckner, 1997), but the Rad55-Rad57 complex has no strand exchange activity (Hays et al., 1995; Johnson and Symington, 1995; Sung, 1997). Their contribution to the stabilization of the Rad51 nucleofilament appears to be performed by counterbalancing the antirecombinase activity of Srs2 (Liu et al., 2011). Other novel yeast *Rad51* paralogs, components of the Shu complex, also named PCSS: *Shu1*, *Shu2*, *Psy3*, and *Csm2*, have recently been identified. Their activity also counteracts the antirecombination function of Srs2 and Sgs1 (Mankouri et al., 2007; Bernstein et al., 2011; Sasanuma et al., 2013).

**FIGURE 3:**
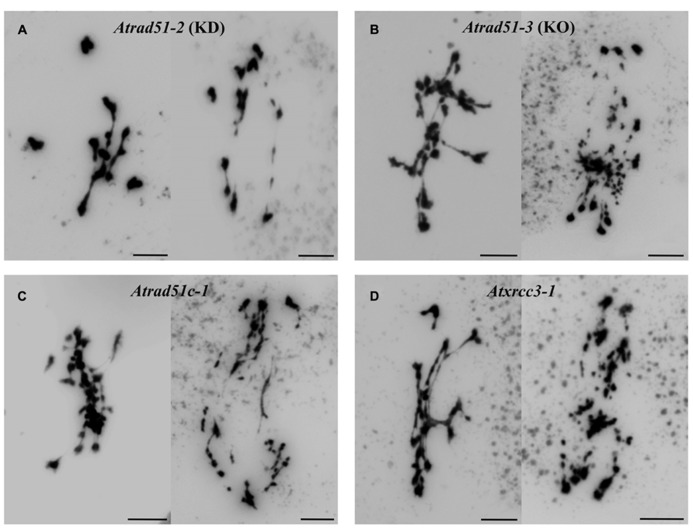
** Some examples of metaphases I and anaphases I in two *Atrad51* mutants and defective mutants for the CX3 complex. (A)** Knockdown (KD) mutant for *Atrad51*: *Atrad51-2*. **(B)** Knockout (KO) mutant for *Atrad51*: *Atrad51-3* (see Pradillo et al., 2012). **(C)** *Atrad51c-1*. **(D)** *Atxrcc3-1*. The meiotic phenotype of *Atrad51-3*, *Atrad51c-1* and *Atxrcc3-1* is quite similar: an entangled mass of chromosomes involving multivalent associations at metaphase I, and missegregations and severe chromosome fragmentation at anaphase I. This phenotype is slighter in the KD mutant *Atrad51-2*: three bivalents and four univalents are clearly distinguished in the picture corresponding to metaphase I. Also, chromosome fragmentation is less drastic at anaphase I. Bars represent 5 μm.

In *Arabidopsis*, *AtRAD51C* and *AtXRCC3* are overexpressed after gamma-irradiation, indicating an essential role for these genes during DNA repair (Osakabe et al., 2002). In addition, the meiotic phenotypes of the corresponding mutants are very similar to that displayed by *Atrad51 *(Bleuyard and White, 2004; Abe et al., 2005; Bleuyard et al., 2005; Li et al., 2005). In this sense, the function of the CX3 complex cannot be replaced by the BCDX2 complex and *Atrad51c* mutation does not produce a more drastic meiotic phenotype than *Atxrcc3*, even though AtRAD51C is present in both complexes (**Figure 3**). In addition, the analysis of *Atrad51c* and *Atxrcc3* mutants in an *Atspo11-1* background has shown that their meiotic chromosomal instability comes from an inability to repair programmed DSBs (Bleuyard et al., 2004; Li et al., 2005). However, there are slightly differences that support the idea that these genes are not functionally identical to *AtRAD51* since the vast majority of *Atrad51*-*1* meiocytes do not have any SCs, whereas in *Atrad51c *there are occasional polycomplexes, and *Atxrcc3 *shows abnormal SCs at a frequency higher than those observed in *Atrad51* (reviewed in Ma, 2006). Hence, *AtRAD51C*, *AtXRCC3*, and *AtRAD51* are not genetically redundant and work together to promote the repair of meiotic DSBs (Bleuyard et al., 2006). In this context, two-hybrid assays performed with *Arabidopsis* sequences confirmed that the CX3 complex interacts with AtRAD51 (Osakabe et al., 2002). For this reason it has been suggested that CX3 facilitates the loading and/or activity of this recombinase during meiotic prophase I.

Roth et al. (2012) have verified that *AtRAD51* and its paralogs *AtRAD51C* and *AtXRCC3* are of great importance for SDSA during somatic recombination. Nevertheless, the exact role of AtRAD51C and AtXRCC3 in plant meiosis and how they collaborate with AtRAD51 to achieve efficient HR remain elusive. Vignard et al. (2007) demonstrated that in the wild-type AtDMC1 loading depends on AtRAD51 and AtXRCC3. However, in a mutant defective for the AtMND1/AtHOP2 complex, which is also essential during early stages of prophase I, AtXRCC3 is dispensable for AtDMC1 focus formation, whereas AtRAD51 is not. Based on this fact, these authors proposed a role for AtXRCC3 in stabilizing AtDMC1 nucleoprotein filaments, since its absence reduces the number of AtDMC1 foci in *Atmnd1*. It is not known whether AtRAD51C, which also carries out an important function during DNA repair in the BCDX2 complex, also presents this function. Furthermore, Da Ines et al. (2012) have analyzed the homologous pairing in *Atrad51*, *Atrad51C*, and *Atxrcc3*, suggesting a separate role for AtDMC1 and AtRAD51-AtRAD51C-AtXRCC3 in synapsis, that should be chromosome region dependent. AtDMC1 would stabilize pairing of homologous centromeric and pericentromeric regions, while AtRAD51 together with AtRAD51C and AtXRCC3 would be necessary for pairing of euchromatic chromosome arms.

With respect to the other paralogs, targeted inactivation of mouse *RAD51B*, *RAD51D*, and *XRCC2* reveals that these genes are essential for mouse embryogenesis (Shu et al., 1999; Deans et al., 2000; Pittman and Schimenti, 2000). In contrast, these genes are not required for viability in *Arabidopsis*, as the triple mutant *Atrad51b Atrad51d Atxrcc2 *shows normal vegetative and reproductive growth (Serra et al., 2013; Wang et al., 2014). However, these mutants are hypersensitive to DNA damaging agents and the genes have partially redundant functions in DNA repair (Bleuyard et al., 2004, 2005; Osakabe et al., 2005; Durrant et al., 2007; Wang et al., 2014). Indeed, the expression of genes involved in both SDSA and single-strand annealing (SSA) pathways is affected in the triple mutant mentioned above. This variation could be a direct consequence of DNA damage, however, the fact that both bleomycin treatment and the triple mutation generate specific sets of differentially expressed genes suggests that each one has a distinct role in gene regulation (Wang et al., 2014). Concerning to this issue Serra et al. (2013) have found that AtXRCC2, AtRAD51B, and AtRAD51D are involved in SSA. Thus, these proteins participate in both RAD51-dependent (SDSA) and RAD51-independent (SSA) HR. Since the meiotic process appears to be normal in the triple mutant *Atrad51b Atrad51d Atxrcc2 *(Wang et al., 2014), the BCDX2 complex seems to be dispensable during meiotic recombination. However, a slight effect should not be ruled out. Indeed, Da Ines et al. (2013b) have reported that the absence of AtXRCC2, and to a lesser extent AtRAD51B, increases rates of meiotic COs. This effect does not occur with AtRAD51D, which is mainly involved in plant immune response (Durrant et al., 2007). Da Ines et al. (2013b) propose that the hyper-recombination phenotype displayed by *Atxrcc2* could be due to an increase in AtDMC1-dependent recombination promoted by a decrease in AtRAD51-dependent recombination.

## OPEN QUESTIONS

Plants, particularly *Arabidopsis*, present apparently relaxed meiotic checkpoint and many mutants, unlike other model organisms, are viable. The exact role of *AtDMC1*, *AtRAD51* and its paralogs in the meiotic HR is still a matter of controversy. The mechanisms through the recombinases cooperate to promote the homology search and the function of *AtRAD51* paralogs in meiosis remain unknown. Although during the last years several studies have contributed to increase the knowledge of the interplay between these proteins, especially by the analysis of the meiotic phenotype of double and even triple mutants, many questions remain unanswered. Are the meiotic defects of *Atrad51* mutants due to the absence of the recombinase or are consequences of a failure in AtDMC1 loading? Does the strand exchange capacity of AtRAD51 play a role during wild-type meiosis or only in certain mutants? Is AtRAD51 able to carry out interhomolog recombination in some genetic backgrounds? Are AtRAD51C and AtXRCC3 functionally equivalent? Are these proteins similarly involved in both intersister and interhomolog recombination? In addition to their early role during meiotic HR, as occur in mammals, do these proteins achieve any activity after strand invasion? Answers to these questions await development of forward-looking molecular tools, generation of new double mutants and finding of new players in this landscape. Time will tell.

## Conflict of Interest Statement

The authors declare that the research was conducted in the absence of any commercial or financial relationships that could be construed as a potential conflict of interest.
